# Outcomes of lung transplantation for scleroderma versus other indications: Insigts from a single center

**DOI:** 10.1016/j.jhlto.2025.100266

**Published:** 2025-04-04

**Authors:** Masashi Furukawa, Ernest G. Chan, John P. Ryan, Chadi A. Hage, Pablo G. Sanchez

**Affiliations:** a Division of Thoracic Surgery, Department of Cardiothoracic Surgery, University of Pittsburgh Medical Center, Pittsburgh PA; b Division of Pulmonary, Allergy and Critical Care Medicine, Department of Medicine, University of Pittsburgh Medical Center, Pittsburgh PA

**Keywords:** Lung transplantation, Scleroderma, Esophageal dysmotility, Gastroesophageal reflux disease

## Abstract

**Background:**

Scleroderma is an autoimmune disease affecting the skin and internal organs, with pulmonary disease being the leading cause of mortality. Lung transplantation is a potential therapy, but its indication has been limited by concerns about complications, such as esophageal dysmotility.

**Methods:**

A retrospective analysis was performed on 959 lung transplant patients from 2011 to 2023, including 77 with scleroderma-related lung disease. Survival rates, post-transplant complications, such as chronic lung allograft dysfunction, and acute cellular rejection rates.

**Results:**

Scleroderma patients had higher mean pulmonary arterial pressure (32 vs. 24 mmHg, p < 0.001) and increased esophageal dysmotility (85% vs. 27%, p < 0.001). Double lung transplantation was more common (99% vs. 87%, p = 0.003). Scleroderma patients experienced higher rates of delayed chest closure (44% vs. 25%, p < 0.001), severe primary graft dysfunction at 72 hours (30% vs. 17%, p = 0.006), and longer mechanical ventilation (median 7 vs. 4 days, p = 0.002). They also required more gastrojejunostomy tubes (79% vs. 20%, p < 0.001) and had longer ICU stays (median 12 vs. 8 days, p = 0.007). However, adjusted competing risks regression showed no significant association between scleroderma and chronic lung allograft dysfunction (HR 0.69 [0.33 – 1.46], p = 0.31) or survival (HR 0.90 [0.56 – 1.45], p = 0.68).

**Conclusions:**

Our findings suggest that lung transplantation might be an important therapeutic option for patients with scleroderma, showing outcomes similar to those of patients with different underlying conditions.

## Introduction

Scleroderma is a systemic autoimmune disease that extensively affects multiple organs, including the skin, lungs, gastrointestinal tract, and the heart.^1^, ^2^ Scleroderma-associated lung diseases, ranging from mild interstitial lung disease to severe pulmonary arterial hypertension (PAH), significantly contribute to morbidity and mortality. Progressive respiratory failure in the lungs is the leading cause of death in patients with scleroderma. Despite therapeutic advances, a subset of patients with advanced scleroderma-related lung disease exhibit refractory lung function decline.^3^, ^4^

Lung transplantation has emerged as a therapeutic option to improve survival and enhance the quality of life in patients with scleroderma.^5^, ^6^, ^7^, ^8^, ^9^ However, concerns regarding disease-specific complications – including gastrointestinal dysmotility, poor nutritional status, and renal disease – have limited the use of lung transplantation in patients with scleroderma.^10^, ^11^, ^12^ Esophageal dysmotility and subsequent aspiration pneumonia are two complications that have been linked to adverse outcomes, such as chronic lung allograft dysfunction (CLAD) and acute cellular rejection in this population.^13^, ^14^

The aim of this study is to identify factors influencing the incidence of acute cellular rejection, development of CLAD, and long-term survival in patients with scleroderma who undergo lung transplantation. We conducted a retrospective analysis of a cohort of patients with scleroderma who underwent lung transplantation at our institution to address the challenges associated with lung transplantation in scleroderma. The primary endpoints were overall survival and CLAD-free survival.

## Patients and methods

### Ethics statement

The study protocol was approved by the Institutional Review Board of the University Pittsburgh (STUDY20050181). The requirement for informed consent was waived because of the retrospective nature of this study.

### Patient population

We conducted a retrospective analysis of lung transplant recipients at the University Pittsburgh Medical Center from January 2011 to March 2023, excluding cases of multiorgan transplant (n = 8) and re-do lung transplants (n = 25). Patient and clinical data were collected by chart review from electronic health records. The diagnosis of scleroderma was based on 2013 American College of Rheumatology/European League Against Rheumatism (ACR/EULAR) classification criteria.^15^ In our study, we classified scleroderma related lung disease into two main subtypes: PAH type and interstitial lung disease (ILD): restrictive subtype. PAH was defined as isolated pulmonary hypertension without significant ILD, while restrictive subtype was classified regardless of the presence or absence of PAH.^4^, ^16^ Right heart catheterization was performed to diagnosed PAH, PAH defined as a resting mean pulmonary artery pressure >25 mmHg.^16^ The primary comparison in all analyses was between patients with a diagnosis of scleroderma and patients with other diagnoses. A supplementary analysis was performed comparing subtypes of scleroderma. We examined the preoperative and operative patient characteristics as well as postoperative outcomes. The primary endpoints were overall survival and CLAD-free survival.

### Statistical methodology

Univariate comparisons were performed using Wilcoxon rank sum tests for continuous variables and Pearson chi-square tests or Fisher exact tests for categorical variables. Nonparametric methods were used due to the imbalance in sample size between the two groups and likelihood of violating assumptions underlying parametric comparisons (e.g. homogeneity of variance in the groups). Post-hoc tests of categorical variables with more than two levels were performed by examining the adjusted standardized residuals of the chi square. Univariable survival analysis for post-transplant survival and survival time to CLAD were performed using the Kaplan-Meier method with a log-rank test. Multivariable analysis for prediction of time-to-CLAD and survival time was performed using Fine and Gray competing risks regression with development of CLAD, retransplant, and death as the competing risks.^17^ Covariates for the multivariable analysis were selected based on clinical experience and pre-transplant group differences.

All statistical analyses were performed using R software (v. 4.4.3). Univariate analyses were performed using *gtsummary* package ^18^ and competing risks analysis was performed with *tidycmprsk*. Values reported are medians unless otherwise noted. A p-value < 0.05 (two-sided) was considered statistically significant for all analyses.

### Upper gastrointestinal evaluation

Esophageal function was evaluated using esophagography with an experienced gastrointestinal radiologist categorizing esophageal dysmotility into four levels: none, mild, moderate, and severe. Moderate-to-severe dysmotility was defined as esophageal dysmotility. Additional tests, such as gastric emptying studies, esophageal impedance studies, 24-hour pH monitoring, and small bowel series, were performed for patients with scleroderma or abnormal screening results, but they were not included in this analysis due to their inconsistent use in all patients.^13^, ^19^

### Postoperative nutrition protocols

After lung transplantation, a nasointestinal feeding tube was placed past the pylorus in the operating room. Enteral feeding started postoperatively with this tube, and oral intake began only after passing the barium swallow test and esophagogram. Patients with moderate to severe esophageal dysmotility required at least three months of being nothing by mouth (*nil per os,* NPO) after lung transplantation. Additionally, they were informed of the necessity of a gastro-jejunostomy (GJ) tube. For patients who already had a gastrostomy tube before transplantation, the tube was transitioned to a GJ tube. For those without a pre-existing gastrostomy tube, a nasointestinal feeding tube was initially inserted in the operating room and positioned beyond the pylorus. A GJ tube was placed during the postoperative recovery phase once the patient's condition stabilized.^13^, ^20^ We used a comprehensive set of tests to assess esophageal motility, swallowing function, and nutritional adequacy to guide the removal of GJ tubes. These included esophageal manometry to evaluate motility, an esophagogram to assess structural integrity, and a swallowing function test to ensure the absence of aspiration risk. A gastric emptying study targeting less than 30% residual volume was also performed to confirm adequate gastric motility. Improvement in parameters such as normalized esophageal motility, effective swallowing without aspiration, and the ability to maintain target weight through oral intake alone guided the decision to remove the GJ tube. For those patients requiring treatment for gastroesophageal reflux disease (GERD), eligible patients had undergone Nissen fundoplication surgery.^19^

## Results

### Patient Demographics and Preoperative Comorbidities

**Figure 1:**
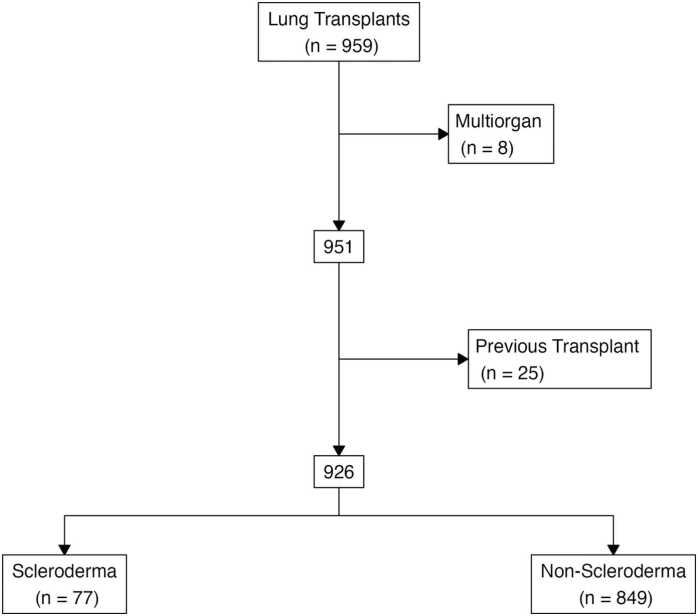
Consort diagram of the study population.

The study included 926 lung transplant recipients; 8 patients with multiorgan transplants and 25 with previous transplants were excluded (Figure 1). Ultimately, 77 were diagnosed with scleroderma and 849 with other conditions (pulmonary fibrosis (45%, n = 382), obstructive disease (35%, n = 295), PAH (2.4%, n = 20), suppurative disease (14%, n = 121). Patients with scleroderma were significantly younger (53 vs. 61 years, p < 0.001) and had a higher proportion of females (68% vs. 39%, p < 0.001) than those in the non-scleroderma group (Table 1). Patients with scleroderma exhibited higher Lung Allocation Scores (LAS) [64 (48−84) vs. 42 (35−63), p < 0.001], higher mean pulmonary pressures [32 (26−44) vs. 24 (20−30) mmHg, p < 0.001], and a higher incidence of esophageal dysmotility [85% vs. 27%, p < 0.001]. Preoperatively, there were no differences between groups in rates of ECMO bridge to transplant (12% vs. 8%, p = 0.26), mechanical ventilator bridge to transplant (12% vs. 9.9%, p = 0.62), or dialysis (p > 0.99).

**Table 1:** Patient Characteristics

		Diagnosis		
Characteristic	N	Non-scleroderma N = 849	Scleroderma N = 77	p-value^a^
Age, Median (IQR)	926	61 (52 – 67)	53 (44 – 59)	<0.001
Sex, n (%)	926			<0.001
Female		335 (39)	52 (68)	
Male		514 (61)	25 (32)	
Body Mass Index (kg/m2), Median (IQR)	926	25.2 (21.3 – 28.9)	25.5 (22.4 – 28.7)	0.66
Lung Allocation Score, Median (IQR)	915	42 (35 – 63)	64 (48 – 84)	<0.001
Mean PA Pressure, Median (IQR)	595	24 (20 – 30)	32 (26 – 44)	<0.001
Esophageal Dysmotility (Preoperative), n (%)	889	219 (27)	64 (85)	<0.001
GERD (Preoperative), n (%)	926	269 (32)	30 (39)	0.19
Preoperative Steroids, n (%)	926	340 (40)	35 (45)	0.35
Liver Disease, n (%)	924	36 (4.3)	4 (5.2)	0.57
Malignancy History, n (%)	926	108 (13)	4 (5.2)	0.052
ECMO Bridge to Transplant, n (%)	926	68 (8.0)	9 (12)	0.26
Mechanical Ventilator Bridge to Tx, n (%)	924	84 (9.9)	9 (12)	0.62
Dialysis (Pre-Transplant), n (%)	923	2 (0.2)	0 (0)	>0.99

ECMO = extracorporeal membrane oxygenation; GERD = gastroesophageal reflux disease; PA = pulmonary artery

a Wilcoxon rank sum test; Pearson's Chi-squared test; Fisher's exact test

### Surgical Procedures and Post-Operative Outcomes

Surgical and post-operative outcomes are displayed in Table 2. Patients with scleroderma had higher rates of double lung transplants (99% vs. 87%, p=0.003). There was a significant association between diagnosis group and type of intraoperative support (p < 0.001). Patients with scleroderma had higher than expected rates of intraoperative ECMO support (44% vs. 27%), and lower rates of no intraoperative support (13% vs. 37%) relative to patients without scleroderma. Rates of intraoperative cardiopulmonary bypass were similar between the two groups (43% vs. 36%). There was no difference between groups in surgical duration (p = 0.20) or intraoperative blood products used (p = 0.64). Following transplant, patients with scleroderma had higher rates of delayed chest closure (44% vs. 25%, p<0.001), and severe primary graft dysfunction (PGD3) incidence at 72 h (30% vs. 17%, p=0.006). There was no difference between the groups in treatment for acute cellular rejection in the first year following transplant (40% vs. 43%, p = 0.57). Mechanical ventilation duration [7 (3−17) vs. 4 (1−12) days, p = 0.002] and ICU stay [12 (5−21) vs. 8 (3−18) days, p = 0.007] were significantly longer in scleroderma patients. Patients with scleroderma also had higher incidence of pneumonia (44% vs. 33%, p=0.045), and a significantly greater need for GJ tubes (79% vs. 20%, p<0.001) relative to those with other diagnoses. The median duration of GJ tube placement was 134 days (range: 59–683 days) in patients with scleroderma.

**Table 2:** Surgical Procedures and Post-Operative Outcomes

	Diagnosis		
Characteristic	Non-scleroderma N = 849	Scleroderma N = 77	p-value^a^
Transplant Type, n (%)			0.003
Double	741 (87)	76 (99)	
Single	108 (13)	1 (1.3)	
Intraoperative Support, n (%)			<0.001
CPB	302 (36)	33 (43)	
ECMO	232 (27)*	34 (44)*	
None	315 (37)*	10 (13)*	
Total Ischemic Time, Median (IQR)	407 (360 – 463)	405 (355 – 460)	0.50
Surgical Duration (hours), Median (IQR)	7.92 (6.67 – 9.12)	8.08 (7.28 – 9.48)	0.20
Total Intraoperative Product Volume (units), Median (IQR)	5 (1 – 12)	5 (2 – 12)	0.64
Delayed Chest Closure, n (%)	211 (25)	34 (44)	<0.001
PGD3 at 72 h, n (%)	132 (17)	22 (30)	0.006
Post-Operative ECMO, n (%)	140 (16)	19 (25)	0.068
Total MV Duration (days), Median (IQR)	4 (1 – 12)	7 (3 – 17)	0.002
Total ICU Stay (days), Median (IQR)	8 (3 – 18)	12 (5 – 21)	0.007
Index LOS (days), Median (IQR)	24 (17 – 38)	27 (20 – 38)	0.13
Dialysis, n (%)	133 (16)	15 (19)	0.38
Stroke, n (%)	11 (1.3)	2 (2.6)	0.30
Reintubation, n (%)	163 (19)	19 (25)	0.25
Ischemic Bowl Requiring Resection, n (%)	26 (3.1)	4 (5.2)	0.30
Hepatic Dysfunction, n (%)	102 (12)	7 (9.1)	0.45
Hemothorax, n (%)	95 (11)	9 (12)	0.89
Pneumonia, n (%)	279 (33)	34 (44)	0.045
Wound Complication, n (%)	148 (17)	17 (22)	0.31
PEG-J Tube, n (%)	164 (20)	60 (79)	<0.001
Bronchial Dehiscence, n (%)	40 (4.7)	4 (5.2)	0.78
Treatment for ACR Within 1 Year, n (%)	336 (40)	33 (43)	0.57
CLAD, n (%)	156 (18)	8 (10)	0.079
One Year Survival, n (%)	687 (86)	61 (80)	0.16
Five Year Survival, n (%)	328 (60)	35 (61)	0.85

* p < 0.05 post hoc

ACR = acute cellular rejection; CLAD = chronic lung allograft dysfunction; CPB = cardiopulmonary bypass; ECMO = extracorporeal membrane oxygenation; ICU = intensive care unit; LOS = length of stay; MV = mechanical ventilation; PEG-J = percutaneous endoscopic gastro-jejunal; PGD3 = primary graft dysfunction grade 3

a Pearson's Chi-squared test; Wilcoxon rank sum test; Fisher's exact test

### Post-transplant survival

**Figure 2:**
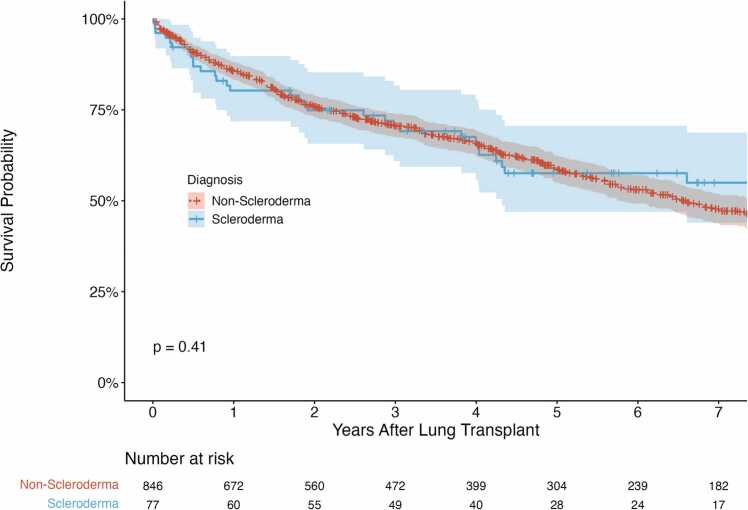
Kaplan-Meier analysis of overall survival. There was not a significant difference in survival time between patients with scleroderma versus other diagnoses (p = 0.41).

**Figure 3:**
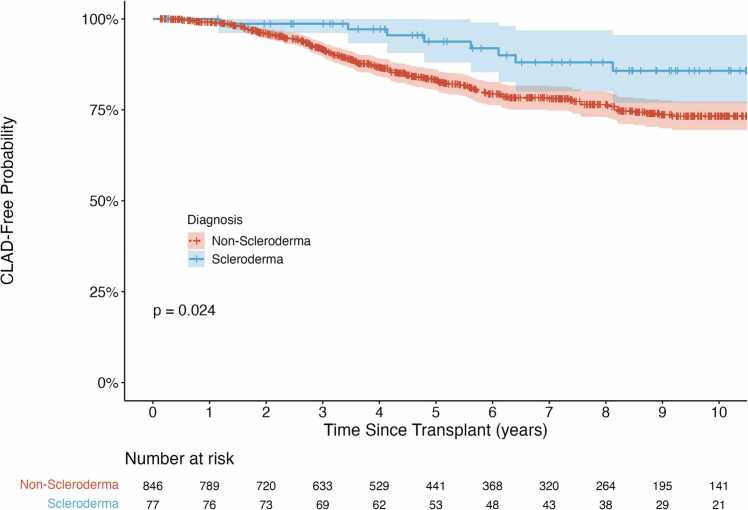
Kaplan-Meier analysis of time-to-CLAD. Patients with scleroderma took significantly longer than other diagnoses to develop CLAD (p = 0.02) in unadjusted analyses.

There was no difference in one-(80% vs. 86%, p=0.16) or five-year survival (61% vs. 60%, p = 0.85) rates between patients with scleroderma and patients with other diagnoses, respectively. Univariable Kaplan-Meier analysis showed no difference in overall survival time between patients with scleroderma and patients with other diagnoses (Figure 2; χ^2^(1) = 0.69, p = 0.41). However, in unadjusted models, patients with scleroderma took longer to develop CLAD relative to patients with other diagnoses (Figure 3; χ^2^(1) = 5.06, p = 0.024).

Multivariable competing risk regression showed no association between scleroderma diagnosis and survival (Table 3; Hazard ratio (HR) 0.90 [0.56 – 1.45], p = 0.68) or CLAD (HR 0.69 [0.33 – 1.46], p = 0.33). Older age was associated with poorer survival (HR 1.02 [1.01 – 1.03], p = 0.002), as well as having a GJ tube placed (1.58 [1.20 – 2.09], p = 0.001). GJ tube placement was also protective against CLAD (HR 0.57 [0.37 – 0.89], p = 0.012). Sex was not associated with survival (HR 1.07 [95% CI: 0.85–1.34], p = 0.56) or chronic lung allograft dysfunction (CLAD) (HR 1.01 [95% CI: 0.74–1.47], p = 0.95) in adjusted analyses. Similarly, esophageal dysmotility showed no significant association with survival or CLAD in the adjusted models. Due to the high degree of missing data (35%) in the mean pulmonary artery pressure covariate, it was not included in the initial multivariable model. To investigate the potential effect of mean pulmonary artery pressure, a separate multivariable regression was performed with mean pulmonary artery pressure as a covariate. Mean pulmonary pressure was not a significant predictor of either survival (HR 0.99 [0.98 – 1.01], p = 0.42) or CLAD (HR 1.01 [1.00 – 1.03], p = 0.11). The significance and direction of other relationships and outcomes did not change with the covariate added to the model (data not shown).

**Table 3:** Adjusted Cumulative Risk Regression of Post-Transplant Outcomes

	CLAD			Death	
Characteristic	N	HR (95% CI)	p-value	HR (95% CI)	p-value
Scleroderma					
Non-Scleroderma	813	—		—	
Scleroderma	75	0.69 (0.33 to 1.46)	0.33	0.90 (0.56 to 1.45)	0.68
Age	888	1.00 (0.98 to 1.01)	0.40	1.02 (1.01 to 1.03)	0.002
Sex					
Female	373	—		—	
Male	515	1.01 (0.74 to 1.38)	0.95	1.07 (0.85 to 1.34)	0.56
Esophageal Dysmotility (preoperative)	888	1.04 (0.74 to 1.47)	0.83	1.01 (0.79 to 1.30)	0.93
PEG-J Tube	888	0.57 (0.37 to 0.89)	0.012	1.58 (1.20 to 2.09)	0.001

Abbreviations: CI = Confidence Interval, HR = Hazard Ratio, PEG-J = percutaneous endoscopic gastro-jejunal

A subgroup analysis was performed to examine potential differences in outcomes within different subtypes of scleroderma. Within the scleroderma group, there were sixty cases of the restrictive type and seventeen cases of the PAH type. Patients with PAH subtype had lower lung allocation scores than those with restrictive subtype (Supplemental table S1; 50 [43–65] vs. 67 [50–85], p = 0.03), but there were no significant differences between subgroups in age (55 [45–61] vs. 53 [44–58], p = 0.54), sex (41% vs. 30% male, p = 0.39), body mass index (25.5 [22.6–29.4], 25.4 [22.3–28.7], p = 0.96), mean PA pressure (41 [40–43] vs. 29,[25–44] p = 0.29), or ECMO bridge-to-transplant between the two groups (0% vs. 15%, p = 0.19). Postoperatively, wound complications were more common in patients with restrictive subtypes (Supplemental Table S2; 28% vs. 0%, p = 0.017), but there were no differences between the groups in bronchial dehiscence (5.9% vs. 5.0%, p > 0.99) or treatment for acute cellular rejection in the first year (41% vs. 43%, p = 0.87). There was also no difference detected in post-transplant survival time (p = 0.87) (Supplemental Figure S1).

## Discussion

Recent studies have suggested similar post-transplant outcomes in patients with scleroderma compared with those with other causes of interstitial lung disease.^7^, ^8^, ^13^, ^14^

In a previous study, 72 patients with scleroderma-related lung disease were compared with 311 patients with interstitial lung disease due to other causes. The study found similar 1- and 5-year survival rates between the two groups. Furthermore, the scleroderma group had a longer period of freedom from CLAD than the other groups in the same timeframe.^13^ One of the major challenges of lung transplantation for scleroderma-related lung disease is the presence of esophageal dysmotility and gastroesophageal reflux disease.^21^, ^22^ Since the issues related to the esophagus are not improved by the lung transplant itself, there is a risk of repeated aspiration pneumonia after the transplant, which may lead to a decline in lung function.^23^ Barium swallow is useful for assessing structural abnormalities and major motility disorders and allows for standardized evaluation across all patients. However, its sensitivity for detecting subtle motility abnormalities is limited.^24^ In contrast, high-resolution manometry provides superior diagnostic accuracy in identifying esophageal motility disorders, including achalasia and ineffective esophageal motility.^21^, ^25^

Since high-resolution manometry was performed selectively in high-risk patients at our institution, we chose barium swallow as the primary method to avoid selection bias in our analysis. However, recognizing the limitations of barium swallow, future studies should use high-resolution manometry more consistently to improve the accuracy of esophageal function assessment. At our institution, the postoperative NPO period is set at a minimum of three months. However, practices vary widely, with some centers, such as Chan et al., extending this period to six months,^23^ while others, like Miele et al., initiating oral intake similarly to protocols for other lung diseases.^14^ Currently, there is no established consensus on the optimal duration of the NPO period.

Our group has previously reported that the presence of a GJ tube after lung transplantation is not associated with survival rates or freedom from CLAD.^19^ Given that the sample of patients is relatively similar between the two reports, the difference in results is possibly due to the difference in methods – the previous report used a balanced sample of patients to explicitly examine the causal association between GJ tube placement and outcomes. The present study, focused on scleroderma, suggests that the association between scleroderma and freedom from CLAD in the univariable analysis was likely due to confounding by the GJ tube placement in the scleroderma group. Although the insertion of a GJ tube is an invasive procedure, it offers benefits such as enabling the drainage of gastric contents, which helps reduce lung injury caused by gastroparesis or silent aspiration. Additionally, it allows for consistent and reliable enteral nutrition. There is a report comparing open gastrostomy and percutaneous gastrostomy in lung transplant patients, which found higher mortality and incidence of acute renal failure in the percutaneous group.^26^ They suggest that open gastrostomy, by directly suturing the stomach to the abdominal wall, reduces the risk of intraperitoneal leakage. In our practice, we use a percutaneous technique to secure the stomach wall.

A multicenter study involving 90 scleroderma patients who underwent lung or heart-lung transplants across 14 European facilities revealed a tendency for lower survival rates in female patients or those with PAH.^8^ However, in the multivariable analysis of our current study, neither female gender nor PAH were significant risk factors for survival. This study showed that the scleroderma group, with a predominant preference for double-lung transplants and a higher incidence of intraoperative support, demonstrated notable differences. These distinctions manifested as increased rates of delayed chest closure, elevated PGD3 levels, prolonged duration of mechanical ventilation, and extended stay in the ICU. As noted in the 2021 ISHLT consensus papers on connective tissue disease and lung transplantation, these findings are consistent with reports that scleroderma patients often have pulmonary hypertension, right ventricular dysfunction, and smaller chest cavity, which complicate surgery and increase the risk of complications.^27^ In our study, the one-year survival rate in the scleroderma group was notably lower, although non-significant, with a 20% mortality rate. This may be related to the higher LAS, prevalence of pulmonary hypertension, and increased rates of severe PGD in the scleroderma group. Although previous studies have established a link between PGD and one-year survival, the lack of significance in the present study is likely due to a lack of statistical power. Future studies with larger cohorts will be needed to identify causal pathways between scleroderma, post-transplant complications and survival.

Delayed chest closure is utilized in lung transplantation when patients face significant challenges such as severe pulmonary edema, hemodynamic instability, coagulopathy, impaired oxygenation, or when oversized grafts are used.^28^ This technique helps mitigate risks associated with primary chest closure, including airway resistance and hemodynamic issues.^29^ In scleroderma patients, the increased use of delayed chest closure likely reflects the complexity of their transplants, particularly due to significant mismatches between donor lung size and recipient lung or chest cavity volumes. Singh's study highlights that these mismatches, identified through CT-based volumetric analysis, were a major factor in the need for delayed chest closure, suggesting that traditional predictive equations may not adequately address the unique challenges of lung transplantation in this population.^30^

Our study has several limitations. First, this study was retrospective; therefore, causality cannot be inferred from the analyses. Second, we reported the results of a single-center analysis which may limit the generalizability of these findings to other institutions with different protocols for post-operative nutrition and patient management. Future multi-center studies will be needed to replicate and expand upon these findings. Third, we lacked long-term postoperative data on gastrointestinal dysmotility (and subsequent aspiration pneumonia, and possibly impaired graft function), nutritional status, and renal disease, and therefore could not examine how these factors affected long-term outcomes. An additional limitation was the relatively small sample size in the subgroup analysis as well as the high proportion of missing data on mean pulmonary arterial pressure which reduced statistical power to detect effects. Additionally, the use of non-parametric tests in the univariable analyses may have slightly lower power than parametric tests.

## Conclusion

Lung transplantation for scleroderma-related lung disease has been shown to have comparable survival rates to lung transplantation for other diseases, which is consistent with our findings. Regarding favorable CLAD-free survival in the scleroderma group, it is possible that long-term NPO using techniques such as gastrojejunostomy prevent silent aspiration. Our findings suggest that lung transplantation is a viable option for patients with scleroderma despite the associated challenges. Further studies with larger sample sizes are necessary to confirm these results and identify potential prognostic factors associated with outcomes in patients with scleroderma undergoing lung transplantation.

## Disclosure

The authors declare no conflicts of interest.

## Institutional review board number and date of approval

STUDY20050181: 6/15/2020.

## Data availability statement

Data available on request. The data underlying this article will be shared on reasonable request to the corresponding author.

## Funding

None.

## CRediT authorship contribution statement

**Conceptualization:** Masashi Furukawa, Chadi A. Hage, Pablo G. Sanchez. **Methodology:** Pablo G. Sanchez. **Investigation:** Masashi Furukawa, Ernest G. Chan. **Data analysis:** Ernest G. Chan, John P. Ryan. **Writing – original draft:** Masashi Furukawa. **Writing – review and editing:** John P. Ryan, Chadi A. Hage, Pablo G. Sanchez. **Funding acquisition:** Pablo G. Sanchez. **Supervision:** Pablo G. Sanchez.

## Declaration of Competing Interest

The authors declare that they have no known competing financial interests or personal relationships that could have appeared to influence the work reported in this paper.

## Footnotes

Appendix A Supplemental data associated with this article can be found in the online version at doi:10.1016/j.jhlto.2025.100266.
