# Supplementary material for: Outcomes of lung transplantation for scleroderma versus other indications: Insigts from a single center
Source: JHLT Open. 2025 Apr 4;8:100266. doi: 10.1016/j.jhlto.2025.100266 (PMC12051709; doi:10.1016/j.jhlto.2025.100266)
Supplement: Supplementary file 1 — Supplemental material [file mmc1.docx]

Supplemental Table S1. Patient Characteristics by Scleroderma Type.

|  | | Scleroderma Subtype | |  |
| --- | --- | --- | --- | --- |
| Characteristic | N | PH  N = 17 | Restrictive  N = 60 | p-value^1^ |
| Age, Median (IQR) | 77 | 55 (45 – 61) | 53 (44 – 58) | 0.54 |
| Sex, n (%) | 77 |  |  | 0.39 |
| Female |  | 10 (59) | 42 (70) |  |
| Male |  | 7 (41) | 18 (30) |  |
| Body Mass Index (kg/m2), Median (IQR) | 77 | 25.5 (22.6 – 29.4) | 25.4 (22.3 – 28.7) | 0.96 |
| Lung Allocation Score, Median (IQR) | 76 | 50 (43 – 65) | 67 (50 – 85) | 0.029 |
| Mean PA Pressure, Median (IQR) | 52 | 41 (40 – 43) | 29 (25 – 44) | 0.29 |
| Esophageal Dysmotility (Preoperative), n (%) | 75 | 13 (76) | 51 (88) | 0.26 |
| GERD (Preoperative), n (%) | 77 | 5 (29) | 25 (42) | 0.36 |
| Preoperative Steroids, n (%) | 77 | 8 (47) | 27 (45) | 0.88 |
| Liver Disease, n (%) | 77 | 1 (5.9) | 3 (5.0) | >0.99 |
| Malignancy History, n (%) | 77 | 1 (5.9) | 3 (5.0) | >0.99 |
| ECMO Bridge to Transplant, n (%) | 77 | 0 (0) | 9 (15) | 0.19 |
| Mechanical Ventilator Bridge to Tx, n (%) | 77 | 0 (0) | 9 (15) | 0.19 |
| ^1^Wilcoxon rank sum test; Pearson's Chi-squared test; Fisher's exact test  ECMO = extracorporeal membrane oxygenation; GERD = gastroesophageal reflux disease, Tx = transplant | | | | |

Supplemental Table S2. Surgical Procedures and Post-Operative Outcomes.

|  | | Scleroderma Subtype | |  |
| --- | --- | --- | --- | --- |
| Characteristic | N | PH  N = 17 | Restrictive  N = 60 | p-value^1^ |
| Transplant Type, n (%) | 77 |  |  | >0.99 |
| Double |  | 17 (100) | 59 (98) |  |
| Single |  | 0 (0) | 1 (1.7) |  |
| Intraoperative Support, n (%) | 77 |  |  | 0.86 |
| CPB |  | 7 (41) | 26 (43) |  |
| ECMO |  | 7 (41) | 27 (45) |  |
| None |  | 3 (18) | 7 (12) |  |
| Total Ischemic Time, Median (IQR) | 70 | 409 (360 – 474) | 405 (352 – 460) | 0.49 |
| Surgical Duration (hours), Median (IQR) | 77 | 7.48 (6.83 – 9.47) | 8.32 (7.33 – 9.58) | 0.20 |
| Total Intraoperative Product Volume (units), Median (IQR) | 76 | 6 (2 – 8) | 5 (2 – 13) | 0.72 |
| Delayed Chest Closure, n (%) | 77 | 6 (35) | 28 (47) | 0.40 |
| PGD3 at 72 hours, n (%) | 73 | 3 (20) | 19 (33) | 0.53 |
| Post-Operative ECMO, n (%) | 77 | 4 (24) | 15 (25) | >0.99 |
| Total MV Duration (days), Median (IQR) | 77 | 8 (3 – 16) | 7 (3 – 18) | 0.95 |
| Total ICU Stay (days), Median (IQR) | 77 | 12 (4 – 21) | 12 (6 – 22) | 0.91 |
| Index LOS (days), Median (IQR) | 77 | 25 (19 – 33) | 28 (21 – 40) | 0.39 |
| Dialysis, n (%) | 77 | 5 (29) | 10 (17) | 0.30 |
| Stroke, n (%) | 77 | 0 (0) | 2 (3.3) | >0.99 |
| Reintubation, n (%) | 77 | 3 (18) | 16 (27) | 0.54 |
| Ischemic Bowl Requiring Resection, n (%) | 77 | 1 (5.9) | 3 (5.0) | >0.99 |
| Hepatic Dysfunction, n (%) | 77 | 1 (5.9) | 6 (10) | >0.99 |
| Hemothorax, n (%) | 77 | 1 (5.9) | 8 (13) | 0.67 |
| Pneumonia, n (%) | 77 | 7 (41) | 27 (45) | 0.78 |
| Wound Complication, n (%) | 77 | 0 (0) | 17 (28) | 0.017 |
| PEG-J Tube, n (%) | 76 | 13 (76) | 47 (80) | 0.75 |
| Bronchial Dehiscence, n (%) | 77 | 1 (5.9) | 3 (5.0) | >0.99 |
| Treatment for ACR Within 1 Year, n (%) | 77 | 7 (41) | 26 (43) | 0.87 |
| CLAD, n (%) | 77 | 2 (12) | 6 (10) | >0.99 |
| One Year Survival, n (%) | 76 | 13 (76) | 48 (81) | 0.73 |
| Five Year Survival, n (%) | 57 | 9 (69) | 26 (59) | 0.51 |
| ^1^Fisher's exact test; Wilcoxon rank sum test; Pearson's Chi-squared test  ACR = acute cellular rejection; CLAD = chronic lung allograft dysfunction; CPB = cardiopulmonary bypass; ECMO = extracorporeal membrane oxygenation; ICU = intensive care unit; LOS = length of stay; MV = mechanical ventilation; PEG-J = percutaneous endoscopic gastro-jejunal; PGD3 = primary graft dysfunction grade 3 | | | | |
