# Supplementary figures and images for: Outcomes of lung transplantation for scleroderma versus other indications: Insigts from a single center
Source: JHLT Open. 2025 Apr 4;8:100266. doi: 10.1016/j.jhlto.2025.100266 (PMC12051709; doi:10.1016/j.jhlto.2025.100266)

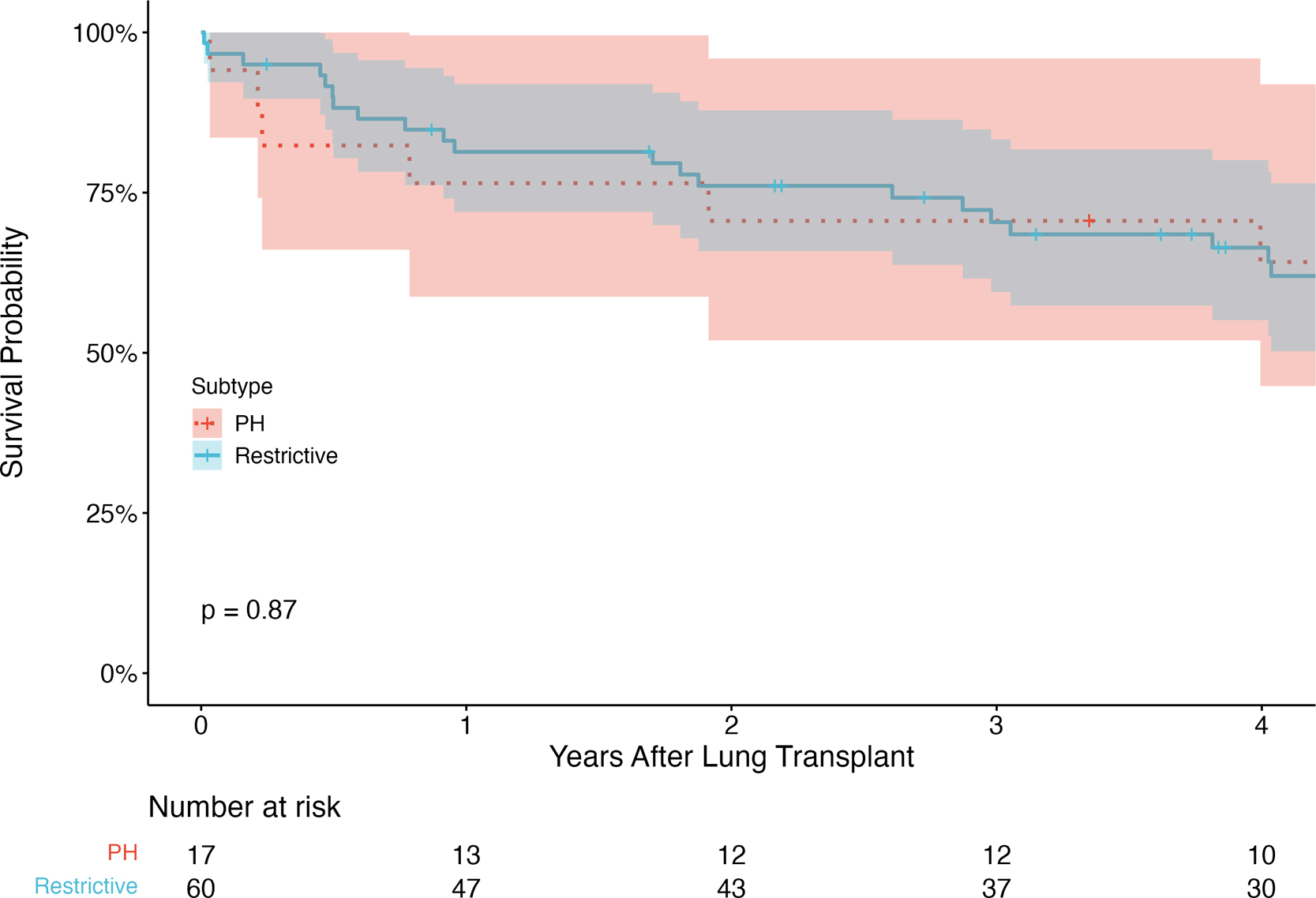

Supplement: Supplementary file 2 — Supplemental material [file mmc2.jpg]
